# Finite element analysis part 2 of 2: Glenohumeral bone stress distribution depends on implant configuration for anatomic and reverse stemless shoulder implants

**DOI:** 10.1002/jeo2.70001

**Published:** 2024-09-19

**Authors:** Victor Housset, Umasuthan Srikumaran, Jean‐Marie Daudet, Léo Fradet, Rohan‐Jean Bianco, Geoffroy Nourissat

**Affiliations:** ^1^ Hôpital Henri‐Mondor, Université Paris‐Est Créteil Créteil France; ^2^ Groupe Maussins, Clinique Maussins Nollete‐Ramsay Santé Paris France; ^3^ Department of Orthopaedic Surgery Baltimore Maryland USA; ^4^ FX Shoulder Solutions Viriat France; ^5^ Philomec Inc. Montréal Québec Canada

**Keywords:** bone stress, finite element analysis, polyethylene cup shape, reverse shoulder arthroplasty, shoulder, stemless, total shoulder arthroplasty

## Abstract

**Purpose:**

Our purpose was to quantify stresses in the bone surrounding stemless implants in various configurations.

**Methods:**

A detailed finite element model of the glenohumeral joint was used to simulate abduction kinematics before and after arthroplasty and to measure bone stresses around the implants. Two digital patients were simulated: one healthy and one with supraspinatus muscle impairment (deficiency). Two anatomic total shoulder arthroplasty (TSA) configurations were placed in a 135° cutting plane. Two reverse shoulder arthroplasty (RSA) configurations with cutting angles of 135° and 145° were simulated with asymmetrical and symmetrical polyethylene cups, respectively, to obtain humeral neck‐shaft angles of 145°.

**Results:**

Compared with preoperative models, TSA preserved and RSA restored abduction kinematics. The bone mechanical stresses were located mainly around the central stud of the TSA and were more peripheral to the RSA humeral components. The RSA configuration with the 145° cutting angle and symmetrical cup generated the lowest maximal bone stress and bone volume involvement. Stresses in the scapular cortical bone were highest in the supraspinatus fossa for TSA and the crest of the acromion for RSA.

**Conclusion:**

Early stability and glenohumeral bone stress change with implant configuration and should not be extrapolated from anatomic clinical data to reverse configurations.

**Level of Evidence:**

Diagnostic tests or criteria; Level IV.

**Abbreviations**
HNSA: humeral neck‐shaft angle
RSA: reverse shoulder arthroplasty
TSA: total shoulder arthroplasty.

## BACKGROUND

The use of stemless anatomic total shoulder arthroplasty (TSA) is growing in surgeons' practices, having demonstrated benefits during more than 20 years of use [28, 30, 40]. The rates of clinical and radiographic failure of stemless TSA are the same as those of stemmed TSA, but in stemless TSA, revision is facilitated by bone preservation [7, 22, 43]. In TSA, stemless implants have been shown to decrease osteolysis, stress shielding, and the risk of periprosthetic fractures and to be safe and effective [23, 42].

Despite having been used for more than 10 years, stemless reverse shoulder arthroplasty (RSA) is less popular [25, 34]. Several reasons are reported: the risk of mobilization, the stress shielding and loosening around the proximal humerus, and the unknown impact of polyethylene cup (i.e., humeral insert) shape on stress transmission [1, 3, 16]. All of these factors may contribute to early loosening of the implant.

Clinical studies have demonstrated the stability of stemless RSA over long‐term follow‐up [7, 8]. However, these studies were retrospective and did not focus on early stability of the implant. Finite element studies have been used to evaluate the primary stability of stemless RSA [13, 36]. Liu et al. [28] demonstrated that stemless RSA had similar clinical and radiological outcomes compared to stemmed RSA for early and mid‐term follow‐ups. They reported that poor bone quality and stability issues anticipated during preoperative planning caused surgeons to choose stemmed implants rather than stemless.

Favre and Henderson [17] evaluated stemless humeral implant micromotion during upper‐limb activities and found that micromotions below 150 μm occurred over at least 99% of the implant surface in all simulated activities. There is a need to consider the full contact interface and physiologic in vivo loading when evaluating primary stability.

Our hypothesis is that glenohumeral bone stress distribution depends on implant configuration. The purpose of this study was thus to better understand the differences in biomechanical behaviour around stemless shoulder arthroplasty and quantify stresses in the bone surrounding stemless implants in various configurations.

## METHODS

### Preoperative model description

We used a previously created and verified finite element model of a glenohumeral joint (paper 1) morphed to the geometric attributes of a healthy male patient (based on a population average) with no known pathologies or deformities [33]. The detailed model included trabecular and cortical bone for the humerus, scapula and clavicle; cartilage and labrum of the glenohumeral joint; glenohumeral ligaments; and 12 muscles involved in scapulohumeral movements, including the rotator cuff and deltoid. The cortical bone volume was created through variable thickening of the bone's external surface. All epiphyses were filled with trabecular bone, and diaphysis were left hollow. The ligament and tendon attachment points were determined from previous studies and confirmed with clinicians [15, 45]. The regional thickness of the cortical bone wall and cartilage was represented according to published morphometric measurements [12, 21, 31, 39].

The model's morphological features (scapular thickness, glenoid version and inclination, glenoid cavity, and head curvature) were compared with published data to ensure that the model represented normal human morphology [11, 27, 32]. The model was meshed with tetrahedral elements of 1 mm for the cartilage and labrum and 2–3 mm for the bone structures, representing characteristic length. A mesh size sensitivity analysis was performed to ensure stability of the model. Spring 1D elements were used to model the ligaments and tendons on the basis of descriptions of their origin and insertion areas. The resulting length of each ligament was similar to measurements taken by Yang et al. [45]. Tendons were modelled using triangular 2D elements. Muscles were modelled with 1D active spring elements with pulling forces specific to each muscle bundle. Thicknesses of each soft‐tissue component were defined individually to maintain an appropriate muscle lever arm relative to corresponding adjacent bones throughout joint motion.

The tendons and ligaments were modelled using linear elastic mechanical properties. Bone structures were modelled using an isotropic elastoplastic material law that represents bone failure by deleting the elements that locally reach a defined failure strain [20].

Abduction motion was performed by applying a linear ramp of increasing force in each muscle element through active stimuli. These forces were calculated according to individual muscle cross‐sectional areas [24] and scaled so that the rotator cuff and middle deltoid forces were 60 and 150 N, respectively [6]. To simulate abduction, we activated only the forces in the middle deltoid, anterior deltoid, subscapularis, supraspinatus, infraspinatus, and teres minor [6, 18, 19]. Muscle activation was increased until 60° of scapulohumeral angle or the maximum deltoid force of 150 N was reached.

The distal half of the scapula was considered a rigid body and its movements fixed in all directions. The distal two‐thirds of the humerus was also considered a rigid body. A vertical force of 25 N was applied to the distal humerus to account for upper‐limb weight [14]. Nonpenetration contact interfaces were defined between the humerus and scapula, as well as between soft tissues and bones. The thickness of each tendon was taken into account in these contact interfaces to provide accurate representation of muscle moment arms [10].

### Configurations

Six simulations were performed using an explicit dynamic finite element solver (Radioss, release 2021.1, Altair Engineering, Inc.).

Two noninstrumented patient configurations were simulated: a healthy patient with an intact rotator cuff and a patient with supraspinatus muscle impairment (‘deficiency’). Deficiency was represented by deactivating forces in the supraspinatus muscle.

**Figure 1:**
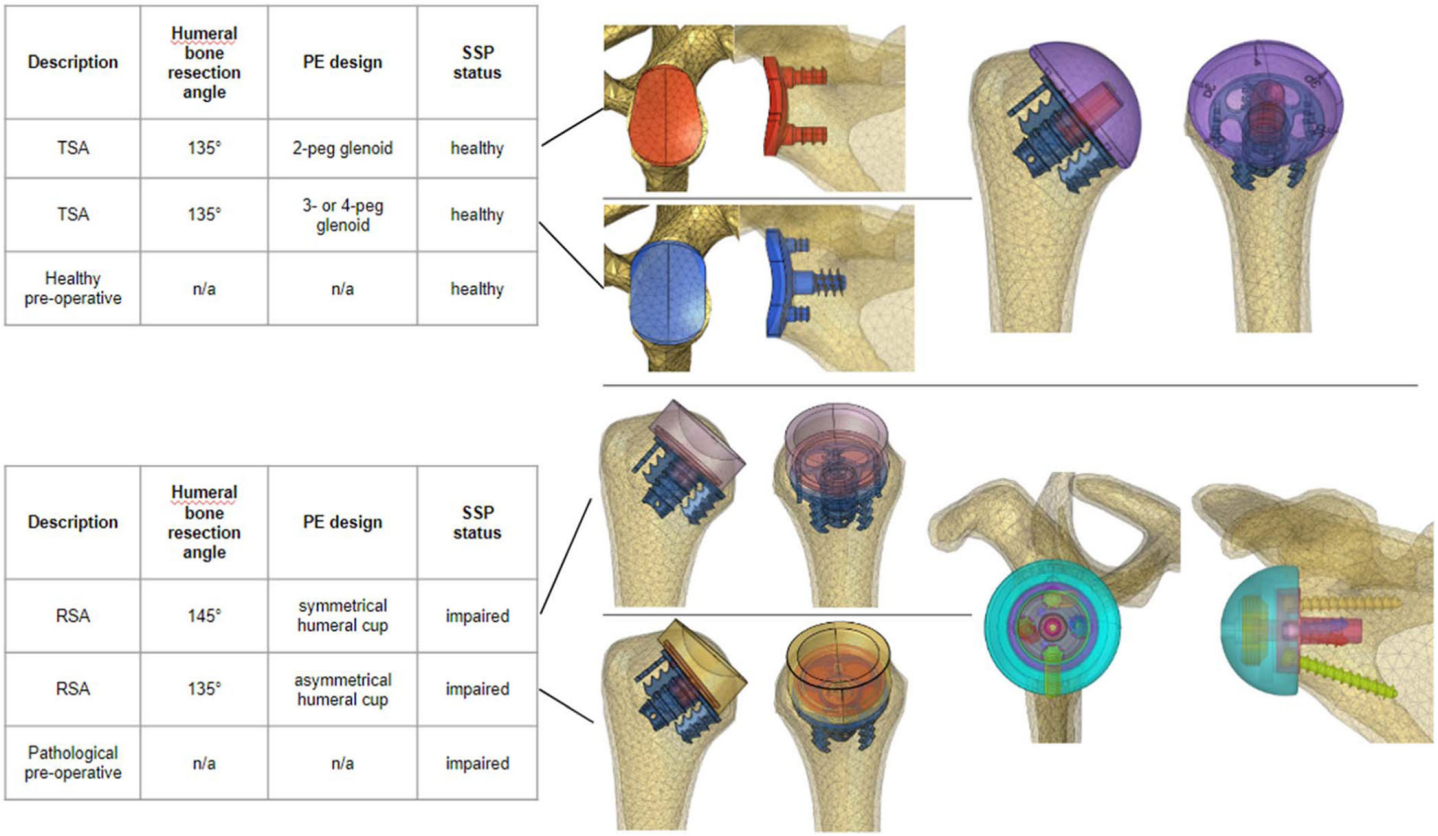
Simulation plan and description of implant placement for the anatomic total shoulder arthroplasty (TSA) configuration (top) and reverse shoulder arthroplasty (RSA) configuration (bottom) and their corresponding glenoid counterparts. PE, polyethylene; SSP, supraspinatus muscle.

Postoperative biomechanics were simulated in these two ‘digital patients’ by using the Easytech Stemless range (FX Shoulder). Four configurations were tested (Figure 1):

- Anatomic TSA in the healthy patient, with a 135° humeral bone cutting plane angle and a 2‐peg glenoid on scapula.
- Anatomic TSA in the healthy patient, with a 135° humeral bone cutting plane angle and a 4‐peg glenoid on scapula.
- RSA in the deficient patient, with a 145° humeral bone cutting plane angle and a symmetrical polyethylene humeral cup, later referred to as 145‐symmetrical cup.
- RSA in the deficient patient, with a 135° humeral bone cutting plane angle and an asymmetrical polyethylene humeral cup, later referred to as 135‐asymmetrical cup.

The last two configurations were defined to obtain identical humeral neck‐shaft angles (HNSAs) of 145°.

Elastoplastic material properties (Johnson–Cook model) for TA6V ELI titanium alloy and chromium cobalt alloy were calibrated using standard mechanical properties presented in ASTM‐F136 and ASTM‐F90, respectively. Elastoplastic material properties for polyethylene were calibrated using dedicated experimental data.

### Virtual surgical procedure

The virtual surgical procedure included removal of ligament during TSA and RSA, removal of bone stock by Boolean operation using pre‐positioned implant geometry, and removal of the posterosuperior cuff for RSA configurations. The stem and baseplate were positioned blindly by two independent senior surgeons (Umasuthan Srikumaran and Geoffroy Nourissat) to obtain the best positioning as described by the manufacturer. A contact interface with friction was applied between the implant and adjacent bone, as well as between the humeral head and glenoid, using a point/surface penalty method with a Coulomb‐type friction coefficient of 0.2 and minimal gap of 0.05 mm [9].

### Measurements

For each configuration, trabecular and cortical bone, as well as implant Von Mises stress fields of the humerus and glenoid were described. Joint kinematics and contact forces were also compared between configurations.

## RESULTS

### Glenohumeral joint kinematics

**Figure 2:**
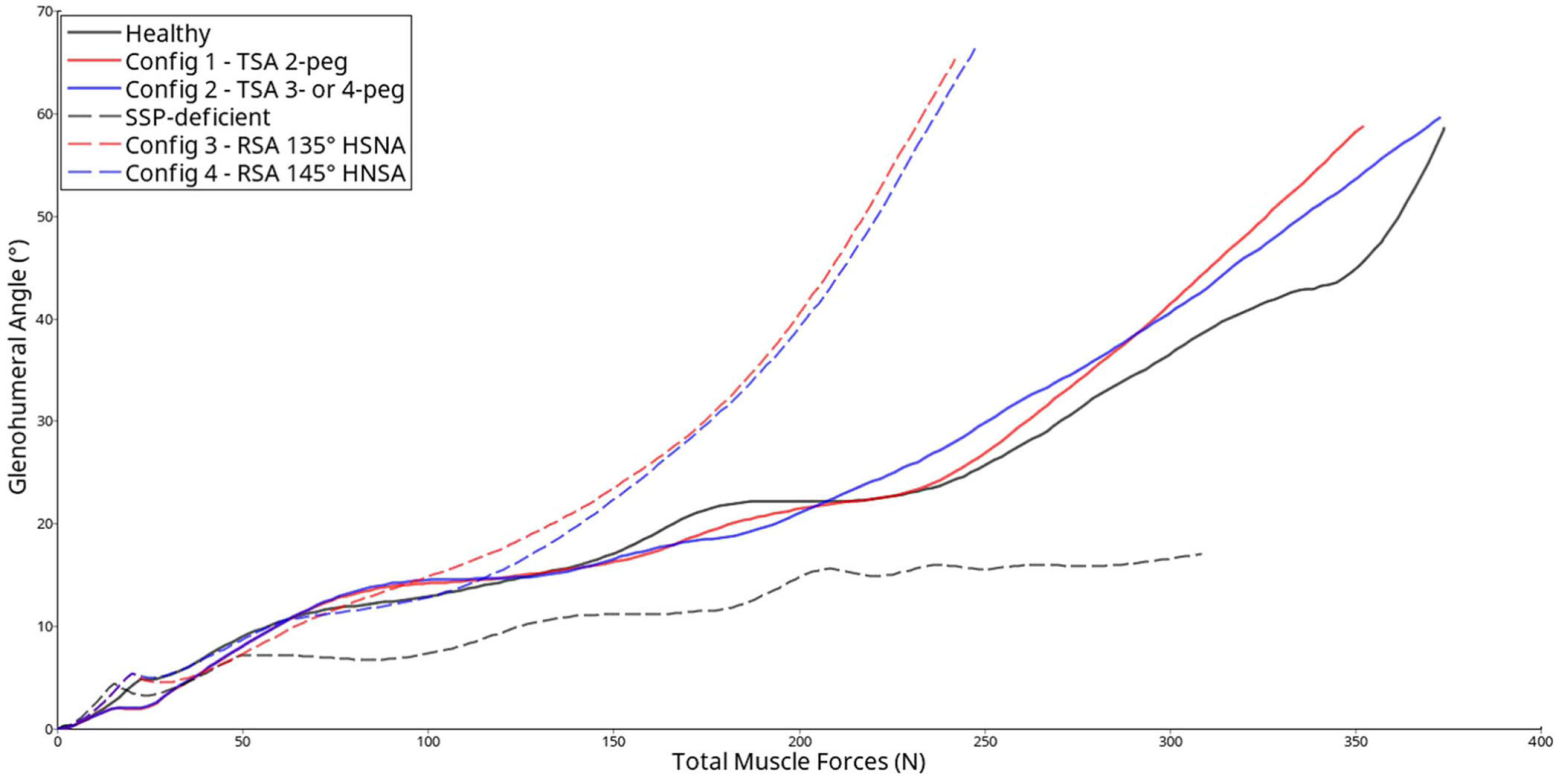
Graph of glenohumeral angle relative to muscle forces for healthy and SSP‐deficient (supraspinatus [SSP]‐impaired) pre‐ and postoperative configurations. The curves show a major improvement of humeral elevation for RSA (dashed curves) compared with preoperative SSP‐deficient simulation and minor differences in kinematics between TSA configurations and preoperative healthy simulation. Config, configuration; HNSA, humeral neck‐shaft angle; RSA, reverse shoulder arthroplasty; TSA, anatomic total shoulder arthroplasty.

The kinematics analysis of abduction in both healthy and deficient models demonstrated that supraspinatus deficiency limits shoulder function, which aligns with clinical expectations. The graph depicting the scapulohumeral angle over time (Figure 2) for the preoperative models illustrates an initial stabilization period from 0 to 50 ms, during which the rotator cuff muscles and the weight of the arm come into play. As the muscles activate, the scapulohumeral angles progressively increase. In the healthy model, a total muscle force of 374 N resulted in a 60° scapulohumeral angle (equivalent to 90° of abduction). In contrast, the deficient model achieved a maximum scapulohumeral angle of only 17° after complete muscle activation (150 N in the middle deltoid).

TSA configurations allowed preservation of the amplitude of abduction with minor modification of the general kinematics compared with the healthy model. The instrumented models showed slightly better kinematics, meaning that at the same level of applied muscle forces, a glenohumeral angle was obtained that was greater than the angle in a healthy native articulation.

We measured only small differences between pre‐ and postoperative abduction kinematics. The main difference was the low abduction angles in the simulations with the healthy model: 55% of muscle forces were needed to reach 20° in the preoperative configuration, whereas only 35% of muscle forces were needed to reach 20° in the postoperative configurations. The anatomic configuration with 4‐peg glenoids showed a more stable kinematic curve, suggesting a more stable sliding of the joint compared with the anatomic configuration with a 2‐peg glenoid. In the supraspinatus deficient model, both RSA configurations successfully restored function in the deficient model, regardless of the shape of polyethylene cup. We observed that the 135° and 145° RSA reached scapulohumeral angles of 53.5° and 55°, respectively, before contact between the humeral greater tuberosity and the acromion.

Compared with anatomic configurations, reverse configurations required less muscle activation to reach a 60° scapulohumeral angle. To achieve a 60° scapulohumeral angle, slightly lower muscle forces were needed with 145‐symmetrical cups than with 135‐asymmetrical cups.

Maximum glenohumeral contact force was higher for anatomic configurations than for reverse configurations. This result was expected because anatomic configurations show less congruence between humeral and glenoid components. Contact forces were higher for anatomic configurations compared to reverse configurations (Table 1).

**Table 1:** Measured contact forces and bone stress at maximum abduction for each simulated configuration.

Configuration	Head‐Glenoid contact force (*N*)	Humerus cortical bone VM stress (MPa)	Scapula cortical bone VM stress (MPa)	Humerus trabecular bone VM stress (MPa)	Scapula trabecular bone VM stress (MPa)
1—TSA; 2 pegs	534	4.9	27.7	2.7	4.1
2—TSA; 4 pegs	506	6.2	29.9	2.6	3.3
3—RSA 135° bone cut	374	3.2	24.3	1.2	3.6
4—RSA 145° bone cut	395	2.7	25.3	1.1	4.6

### Humerus stress analysis

**Figure 3:**
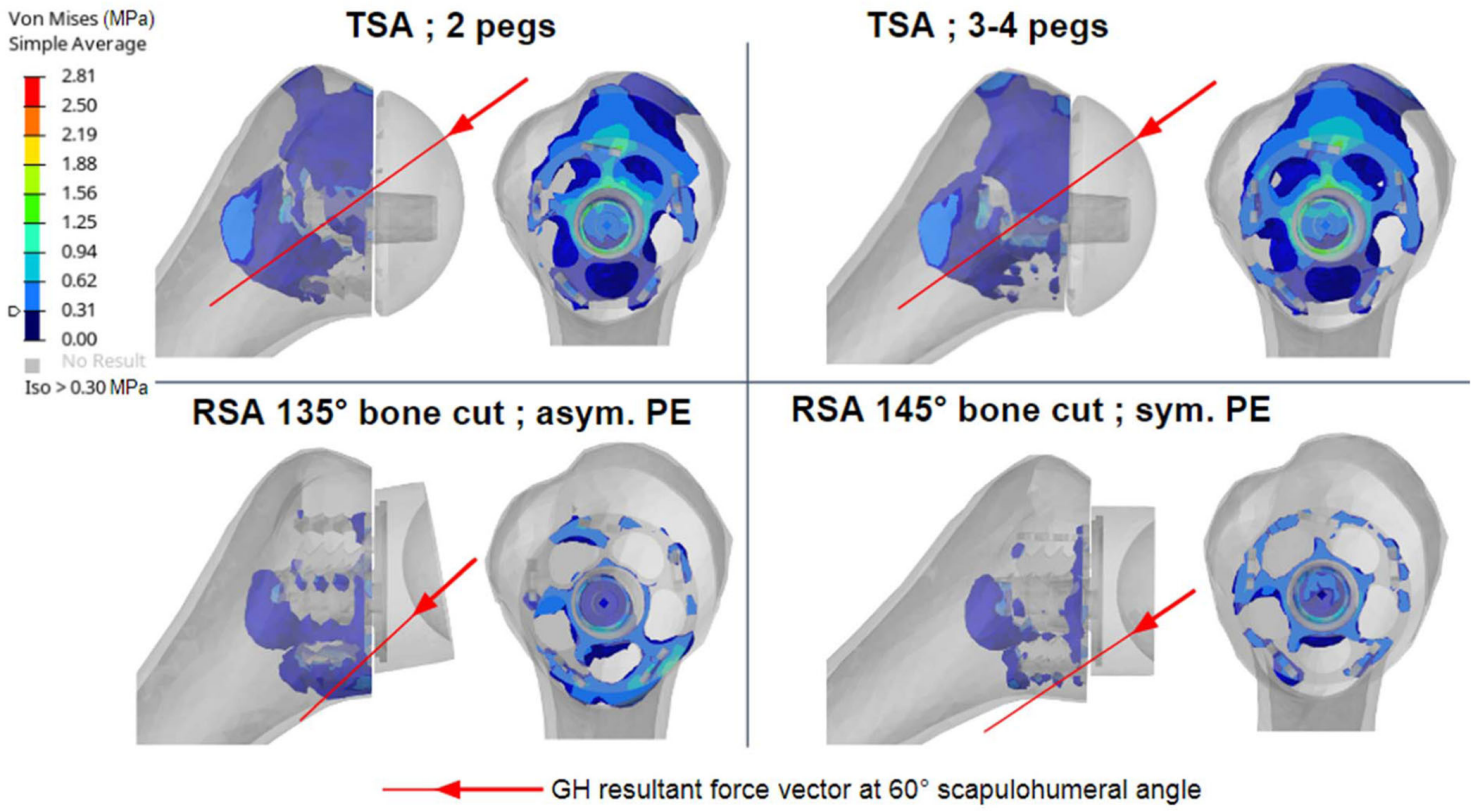
Glenohumeral reaction force directions at 60° abduction angle and resulting peri‐implant humeral trabecular bone stress. Trabecular areas where Von Mises stresses are greater than 0.3 MPa are highlighted in colour. asym., asymmetrical; GH, glenohumeral; PE, polyethylene; RSA, reverse shoulder arthroplasty; SH, scapulohumeral; sym., symmetrical; TSA, anatomic total shoulder arthroplasty.

**Figure 4:**
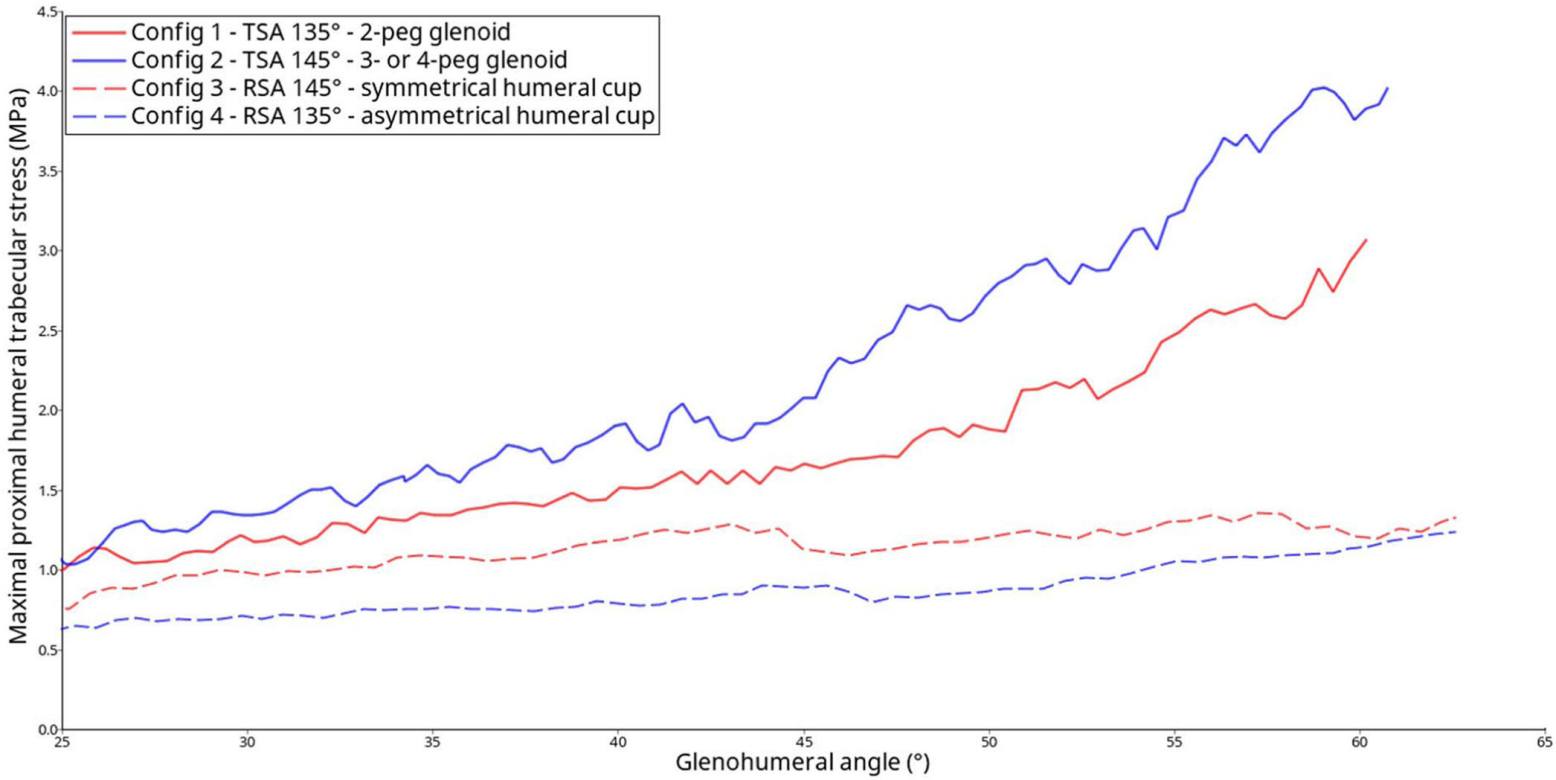
Maximal proximal humeral trabecular Von Mises stress relative to glenohumeral elevation for all postoperative configurations during abduction motion. Config., configuration; RSA, reverse shoulder arthroplasty; TSA, anatomic total shoulder arthroplasty.

The bone mechanical stresses were located mainly around the central stud of the anatomic component and were more peripheral in the two reverse configurations (Figure 3). The RSA configuration with the 145° cutting angle and symmetrical cup generated lower maximal bone stress and less bone volume involvement than the 135° cutting angle with the asymmetrical cup did (Figure 4). All maximal humeral trabecular and cortical bone stresses measured were lower than the material limits (65 and 175 MPa, respectively). The TSA configurations led to more stressed volume and higher maximal measured stress compared with the reverse configurations, which was attributable to an active supraspinatus muscle. The main stressed area was located distal to the anchor central peg.

### Scapula stress analysis

**Figure 5:**
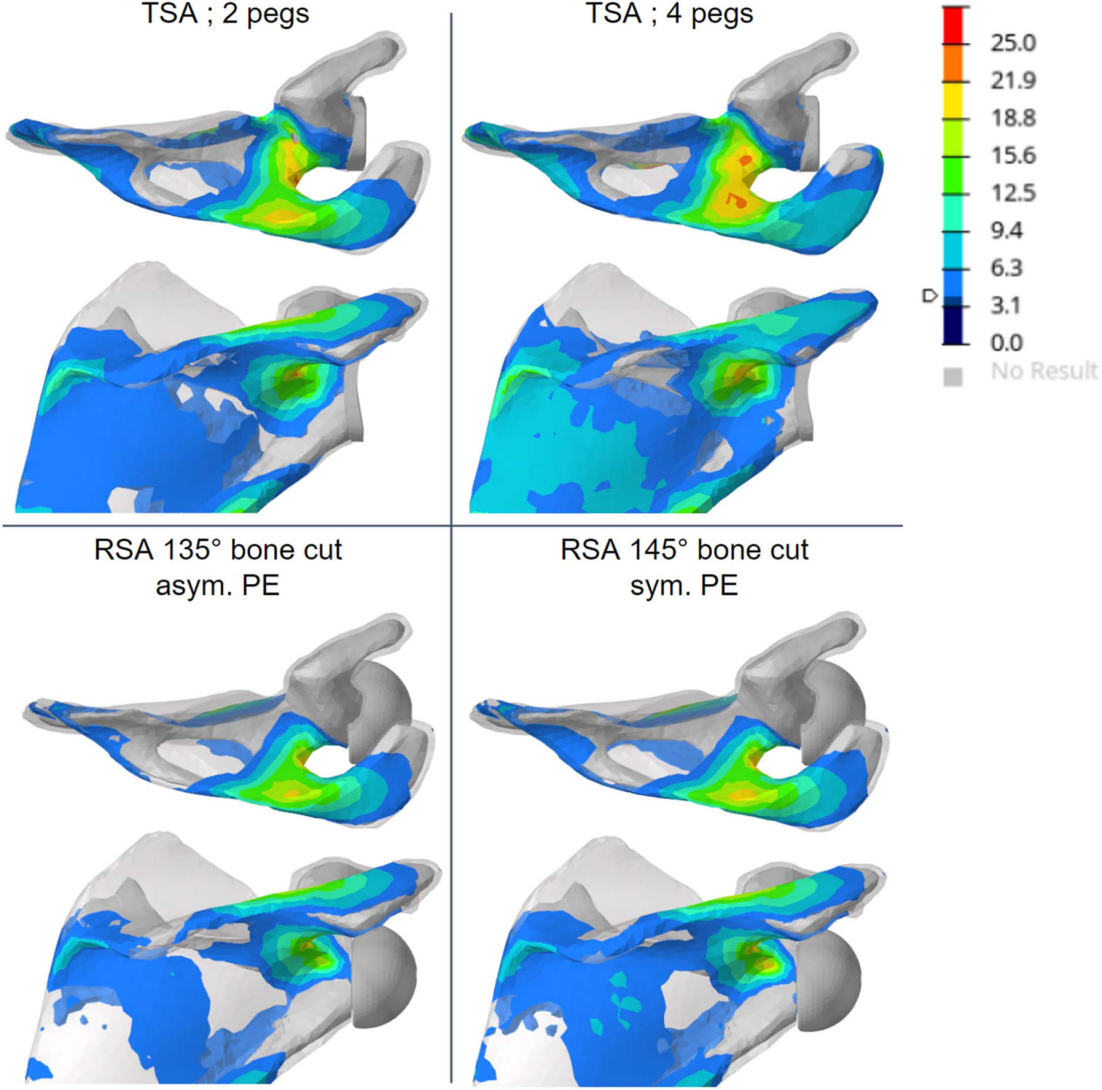
Scapular cortical bone stress at 60° glenohumeral angle (displayed only when exceeding 4 MPa). Superior and posterior views. asym., asymmetrical; PE, polyethylene; RSA, reverse shoulder arthroplasty; sym., symmetrical; TSA, anatomic total shoulder arthroplasty.

Stresses in the cortical bone of the scapula were highest in the supraspinatus fossa for anatomic configurations and near the crest of the acromion for reverse configurations (Figure 5). All maximal scapular trabecular and cortical bone stresses were lower than the material limits (65 and 175 MPa, respectively). The TSA configurations led to more stressed volume and higher maximal measured stress compared with the RSA configurations because of an active supraspinatus muscle and the very different geometry compared with the reverse configurations. Trabecular bone stress was higher for instrumented configurations compared with preoperative models. We found no low‐stress areas in the instrumented models that were stressed in the uninstrumented models, suggesting a low likelihood of stress shielding in reverse configurations. The highest stress in the cortical bone was at the base of the acromion.

### Implant stress

All polyethylene component stresses were below the material limit (22 MPa). Stress was higher in TSA configurations, which showed less congruence between humeral and glenoid components, possibly leading to earlier material wear compared with RSA configurations. The chromium cobalt head stresses were below the material limit (585 MPa). Higher Von Mises stress was measured in the heads in the TSA configurations compared with glenospheres in RSA configurations. Maximal measured Von Mises stresses were lower than material yield strength (795 MPa). The highest stresses were observed around the taper‐anchor contact. The maximum stress at 90° abduction was not the highest stress for the RSA configurations. This finding suggests that the titanium alloy's components' ‘worst‐case’ loading is at an abduction angle of less than 90°.

## DISCUSSION

This finite element model study confirmed our hypothesis that the stresses in the bone surrounding stemless shoulder arthroplasty implants vary depending on the implant configuration.

In the anatomic configuration, the stress is well distributed all around the stemless implant. In the reverse configuration, most of the stress is located in the medial aspect of the proximal humeral trabecular bone, highlighting the role of the calcar integrity in stemless RSA, and potentially partially explaining the process of valgus early migration in cases of poor primary fixation in weak bone. This stress distribution can be explained by specificities of the glenohumeral contact force vector illustrated in Figure 3. Several authors have reported the importance of evaluating bone quality before performing RSA [35]. In a finite element study, Reeves et al. [37] evaluated multiple generic stemless implants with various fixation features to assess the effect of the shape of these implants on simulated stress and strain response of the proximal humerus. They concluded that centrally pegged implants had the lowest simulated resorbing potential, but stemless implants produced the greatest percentage of implant bone contact area. The Easytech Stemless implants (anatomical and reversed) used in the finite element study are designed with a central stud and peripheral fixation [34]. The current study appears to emphasize the importance of this peripheral fixation for immediate stability of the implant.

In RSA stemless configuration, the stress is mostly located in peripheral part of the implant and mostly medial. In anatomic TSA, the stress is centered on the central stub, probably decreasing the importance of peripheral fixation in this configuration. In both humeral and scapular stress analysis, we found that TSA configurations led to more stressed volume and higher maximal measured stress compared with RSA configurations, probably because of an active supraspinatus muscle and the very different geometric properties compared with reverse configurations. In a biomechanical and cadaveric study using musculoskeletal modelling, Ackland et al. [2] compared stemmed TSA and RSA and found that RSA models without supraspinatus muscles produced less joint compression than TSA models with supraspinatus muscles did, which is consistent with our results. In a similar study, the same authors found that RSA with an intact or isolated supraspinatus‐deficient rotator cuff produced large glenohumeral joint forces that may increase baseplate failure risk, particularly during flexion, when posterior shear forces are largest, without a significant difference between the configurations [1]. Conversely, an infraspinatus tear reduced glenohumeral joint compression but also reduced joint stability.

In the current study, the anatomic configuration with the 4‐peg glenoid showed a more stable kinematic curve, suggesting a more stable sliding of the joint compared with the anatomic configuration with a 2‐peg glenoid. Wahab et al. [41] performed a finite element study to investigate the focal stress distribution and relative micromotion between various implant designs with different numbers of pegs and found that implants with four pegs had lower stress critical volume on cement compared with implants with fewer pegs, despite more preserved bone stock. This finding may be more evidence in favour of 4‐peg implants.

There is no consensus regarding the best final HNSA [29]. The current implant is at a HSNA of 145°. In an unpublished study currently under review (paper 1), we demonstrated that modification of HNSA into the polyethylene cup on a short stem does not increase humeral stresses during abduction but results in different ranges of motion and stresses in the metal structures of the implant. The current study shows that for the same 145° HNSA, having a 135° or 145° proximal humeral cut with asymmetric or symmetric polyethylene cup influences the stress around the stemless implant, with higher stress in 135° with an asymmetric polyethylene cup. This finding suggests that the final angle induces different potential for stress‐shielding according to the orientation of the HNSA cut. Clinically, this suggests that in patients with good quality bone, any configuration can be retained, but in patients with weak bone, 145° with a symmetric polyethylene cup should be preferred to decrease the risk of loosening.

Our study also demonstrates that RSA designs induce higher stress in the acromion in ‘zone 2’ as described by Wong et al. [44] in a previous finite element analysis. They also found that an inferior and medial positioning of the glenosphere decreased acromial strain and stress by providing greater deltoid mechanical advantage. In another finite element study, Shah et al. [38] reported several factors as being responsible for scapular stress fracture, such as deltoid lengthening or a more posteriorly oriented acromion. Original RSA designs (Grammont type) were associated with an acromial fracture rate of approximately 2%; however, more recent designs show acromial fracture rates of up to 5% [26, 35, 43].

The current finite element model focuses on immediate stress after implantation. Further studies should consider osseointegration at the bone–implant interface, which would likely influence stress distribution. Also, description of Von Mises stress was achieved only at 90° abduction, which was satisfactory because simulations have shown that trabecular bone stress distribution does not change during abduction movement. In the future, taking into account history of bone stress throughout the motion will provide more information regarding bone growth and degeneration. For this study, we chose to focus on pre‐osseointegration biomechanics because clinical studies report that loosening or migration typically occurs soon after implantation [44, 45]. To our knowledge, no case has been reported of migration occurring after 3 months of good fixation [4, 5].

## CONCLUSION

Early stability and glenohumeral bone stress change with configurations and, in contrast with stemmed implants, the current study suggests that humeral and acromial stress should be evaluated clinically in both anatomic and reverse conditions. Stress distribution around the stemless implant changes in the humerus, so it is not recommended to extrapolate anatomic clinical data to reverse configurations. The orientation of the humeral cut and the shape of the PE influences the peripheral stress around the stemless, suggesting that primary stability of the stemless RSA should be influenced by each factor. Peripheral stability of stemless implant seems mandatory to prevent migration in stemless RSA configuration.

## AUTHOR CONTRIBUTIONS

Victor Housset developed the methodology, wrote the original draft, and revised the manuscript. Umasuthan Srikumaran supervised the study, validated the results, and revised the manuscript. Jean‐Marie Daudet conceptualized the study, developed the methodology, managed the project, and provided resources. Léo Fradet and Rohan‐Jean Bianco performed statistical analyses, developed the methodology, programmed the software, supervised the project, and wrote the original draft. Geoffroy Nourissat conceptualized the study, developed the methodology, and revised the manuscript.

## CONFLICT OF INTEREST STATEMENT

Philomec received financial compensation for their services from FX Shoulder Solutions related to the subject of this article. As a responsible and ethical consulting firm, Philomec has made every effort to maintain the integrity of the article and to avoid any bias that may have been introduced due to this financial relationship. Dr. Umasuthan Srikumaran is a board/committee member of the American Academy of Orthopaedic Surgeons, the American Shoulder and Elbow Surgeon, and the Indian American Shoulder & Elbow Surgeons; holds stock/stock options for ROM3, Sonogen, and Tigon Medical; is a paid consultant for Fx Shoulder and Tigon Medical, receives other financial/material support from Arthrex and DePuy; receives publishing royalties from Thieme; and receives IP royalties from Fx Shoulder and Tigon Medical. The authors declare no conflict of interest.

## ETHICS STATEMENT

Not applicable.

## Data Availability

Due to commercial restrictions, supporting data are not available.
